# Thermodynamics in the Universe Described by the Emergence of Space and the Energy Balance Relation

**DOI:** 10.3390/e21020167

**Published:** 2019-02-11

**Authors:** Fei-Quan Tu, Yi-Xin Chen, Qi-Hong Huang

**Affiliations:** 1 School of Physics and Electronic Science, Zunyi Normal University, Zunyi 563006, China; 2 Zhejiang Institute of Modern Physics, Zhejiang University, Hangzhou 310027, China

**Keywords:** emergence of space, first law, entropy

## Abstract

It has previously been shown that it is more common to describe the evolution of the universe based on the emergence of space and the energy balance relation. Here we investigate the thermodynamic properties of the universe described by such a model. We show that the first law of thermodynamics and the generalized second law of thermodynamics (GSLT) are both satisfied and the weak energy condition are also fulfilled for two typical examples. Finally, we examine the physical consistency for the present model. The results show that there exists a good thermodynamic description for such a universe.

## 1. Introduction

Numerous astro-observations show that our universe is in accelerating expansion at present [1,2]. There are usually two ways to explain the phenomenon if we consider the evolution of the universe from the point of view of the dynamics of gravity. One is the modification of geometric part of Einstein’s field equation, such as f(R) theory, Lanczos-Lovelock gravity theory. The other is the modification of the material part of Einstein’s field equation by introducing the extra matter with negative pressure or the scalar field (called dark energy). A good model which can explain the current accelerated expansion of the universe is ΛCDM (lambda cold dark energy) model which considers a cosmological constant, i.e., the value of the equation of state parameter ω=−1 [3,4]. However, astronomical observations allow also ω to vary with time. The variation with time is described usually by the slowly varying scalar field such as the quintessence [5,6,7,8,9], kinetic energy driven *k*-essence [10,11,12] and tachyon [13,14,15,16,17]. These models describe the accelerated expansion of the universe well.

Studying gravity from a thermodynamic point of view is an interesting field in modern theoretical physics. The deep connection between gravity and thermodynamics is accepted generally because of the black hole thermodynamics [18,19,20] and AdS/CFT correspondence [21]. The equivalence between the Clausius relation δQ=TdS (which connects the heat flow δQ, entropy *S* and Unruh temperature *T*) and the Einstein equation was first given by Jocobson in 1995 [22]. Although the topology of quasi-de Sitter apparent horizon is quite different from that of the local Rindler horizon, the Friedmann equation with the slow-roll scalar field can be reproduced by using the first law of thermodynamics −dE=TdS where dE is the amount of the energy flow through the quasi-de Sitter apparent horizon [23]. Besides, the Friedmann equation can also be derived by calculating the heat flow through the horizon in an expanding universe and by applying the Clausius relation to a cosmological horizon [24]. Padmanabhan [25,26,27,28], in the reverse way, showed that gravitational field equations in a wide variety of theories can reduce to the thermodynamic identity TdS=dE+PdV when evaluated on a horizon. These conclusions further reveal the relation between horizon thermodynamics and spacetime dynamics. Furthermore, Padmanabhan [29,30] revealed the relation between the degrees of freedom of space and the dynamic evolution of the universe. He derived the standard Friedmann equation of the FRW universe through a simple equation $\Delta V=\Delta t\left(N_{sur}-N_{bulk}\right)$, where *V* is the Hubble volume in Planck units, *t* is the cosmic time in Planck units, $N_{sur}$ is the number of the surface degrees of freedom $N_{sur}$ and $N_{bulk}$ is the number of the bulk degrees of freedom. Namely, the difference between the number of the surface degrees of freedom and the number of the bulk degrees of freedom in a region of space drives the accelerated expansion of the universe.

From the point of view of thermodynamics, it tends to spontaneously thermodynamic equilibrium for an isolated thermodynamical system. That is to say, the entropy of an isolated thermodynamical system, *S*, cannot decrease and reaches its maximum finally. In black hole physics, there exists a similar law which is called as the generalized second law of thermodynamics [19]. The law states that the sum of the entropy of the black hole horizon, $S_{h}$, plus the entropy of the matter, $S_{m}$, cannot decrease with time, i.e., $\dot{S}={\dot{S}}_{m}+{\dot{S}}_{h}\geq 0$ where the dot denotes the derivative with respect to time. After that, the generalized second law of thermodynamics (GSLT) was extended to the cosmological horizons [31,32]. In the cosmological context, the GSLT has been extensively studied (see, for example, [33,34,35]).

In Ref. [36], we considered the current accelerated expansion of the universe based on the emergence of space and the energy balance relation $\rho V_{H}=TS$, where ρ is the energy density of the cosmic matter, $S=A_{H}/\left(4L_{p}^{2}\right)=\pi H^{-2}/L_{p}^{2}$ is the entropy associated with the area of the Hubble sphere $V_{H}=\frac{4\pi }{3H^{3}}$ and TS is the heat energy of the boundary surface. Then we found that the evolution solutions of the universe include the solutions obtained from the standard general relativity theory, and concluded that it is more common to describe the evolution of the universe in the thermodynamic way. Therefore, it is interesting to investigate whether the first law of thermodynamics and the GSLT hold in the model described by Ref. [36].

The goal of the present paper is to study the thermodynamical behavior of the universe considered in Ref. [36] by means of its description of the emergence of space and the energy balance relation. Our analysis shows that the first law of thermodynamics holds and is actually the Clausius relation. The validity of the Clausius relation means that the evolution of the universe can be deemed as a series of quasistatic processes. We also show that the GSLT holds in the total accelerated evolutionary history of the universe and the total entropy of the universe tends to the maximal value when the universe evolves to the de Sitter universe by considering two typical examples. The results show that there exists a good thermodynamic description for such a universe.

The paper is organized as follows. In Section 2, we briefly review the model which describes the evolution of the universe based on the emergence of space and the energy balance relation. In Section 3, we show that the first law of thermodynamics holds in the universe descried by the present model. In Section 4, the validity of the GSLT and thermodynamic equilibrium are shown; we also obtain the constraints imposed on the energy density and the pressure of the matter. Our conclusions are presented in Section 5. We use units c=ℏ=1.

## 2. Dynamical Evolution Equations of the Universe Based on the Emergence of Space and the Energy Balance Relation

Let us begin with the FRW metric which describes the homogeneous and isotropic universe

(1) $$ds^{2}=-dt^{2}+a^{2}\left(t\right)\left(\frac{dr^{2}}{1-kr^{2}}+r^{2}d\Omega^{2}\right)=h_{ab}dx^{a}dx^{b}+R^{2}d\Omega^{2},$$

where R=a(t)r is the comoving radius, $h_{ab}=diag\left(-1,\;\frac{a^{2}}{1-kr^{2}}\right)$ is the metric of 2-spacetime ($x^{0}=t,\;x^{1}=r$) and k=0,±1 denotes the curvature parameter. Padmanabhan [29,30] thought that our universe is asymptotically de Sitter and its evolution can be described by the following law

(2) $$\frac{dV}{dt}=L_{p}^{2}\left(N_{sur}-N_{bulk}\right),$$

where $L_{p}$ is the Planck length,

(3) $$N_{sur}=\frac{4\pi }{H^{2}L_{p}^{2}}$$

is the number of the surface degrees of freedom on the Hubble horizon in which *H* is the Hubble constant,

(4) $$N_{bulk}=\frac{\left|E\right|}{\left(1/2\right)k_{B}T}=-\frac{2(\rho +3p)V}{k_{B}T}$$

is the number of the bulk degrees of freedom in which *T* is the temperature of the horizon, $k_{B}$ is the Boltzmann constant, |E|=|(ρ+3p)V| is the Komar energy and $V=\frac{4\pi }{3H^{3}}$ is the Hubble volume. The law (2) indicates that the difference between $N_{sur}$ and $N_{bulk}$ drives the universe towards “holographic equipartition” (i.e., $N_{sur}=N_{bulk}$).

According to the analysis of Ref. [36], the temperature of Hubble horizon of the flat FRW universe is employed as

(5) $$T=\frac{H}{2\pi }\left(1+\frac{\dot{H}}{2H^{2}}\right).$$

Here, we assume $1+\frac{\dot{H}}{2H^{2}}>0$. The study of quantum field theory in a de Sitter space [37] showed that a freely falling observer would measure a temperature T=H/2π on the de Sitter horizon when the radius of the de Sitter horizon is taken as 1/H. Our universe is asymptotically de Sitter, so the temperature of the Hubble horizon should tend to H/2π when *t* becomes large enough. In fact, the approximation $|\dot{H}/2H^{2}|\ll 1$ has been used in calculating the energy flow crossing the apparent horizon [23,24,38,39]. Therefore, it seems to be reasonable to assume $1+\frac{\dot{H}}{2H^{2}}>0$ when we investigate the thermodynamic properties and dynamical behavior of the accelerated universe. In Section 4, we will show the validity of the assumption $1+\frac{\dot{H}}{2H^{2}}>0$ by two typical examples.

Thus, inserting Equations (3)–(5) into Equation (2), we obtain the Friedmann acceleration equation

(6) $$\frac{\ddot{a}}{a}=H^{2}+\dot{H}=-\frac{4\pi L_{p}^{2}}{3}\left(\rho +3p\right)-\frac{\dot{H}}{2}-\frac{\dot{H^{2}}}{2H^{2}}.$$

On the other hand, according to the energy balance relation $\rho V_{H}=TS$ [40], we obtain another evolution equation of the universe

(7) $$H^{2}=\frac{8\pi L_{p}^{2}}{3}\rho -\dot{\frac{H}{2}}.$$

Combining Equations (6) and (7), the following equation

(8) $$\dot{\rho }+3H\left(\rho +p\right)=\frac{3}{8\pi L_{p}^{2}}\left(\frac{\ddot{H}}{2}-\frac{{\dot{H}}^{2}}{H}\right)$$

can be obtained. In this way, we obtain the dynamical evolution equations of the universe based on the emergence of space and the energy balance relation. It was shown [36] that it is more common to describe the evolution of the universe in such a thermodynamic way because the solutions of the dynamical evolution equations in such a model include the solutions obtained from the standard general relativity theory.

## 3. First Law of Thermodynamics for the Present Model

Now that the dynamical evolution of the universe based on the emergence of space and the energy balance relation has been investigated in Ref. [36], it is natural to ask whether the thermodynamic properties (the first law of thermodynamics and the GSLT) can hold in such a model. Furthermore, we may ask what are the constraints on the evolution of the universe if the GSLT holds. In this and the next sections, we will discuss the first law of thermodynamics and the GSLT in such a model respectively.

The study of quantum field theory in a de Sitter space [37] showed that a freely falling observer would measure a temperature T=κ/2π on the de Sitter horizon where κ is the surface gravity. For the *Q* space, Bousso [41] argued its thermodynamical description and showed that the first law of thermodynamics −dE=TdS holds on the apparent horizon. Furthermore, Cai and Kim [38] derived the Friedmann equation of the FRW universe with any spatial curvature based on the first law of thermodynamics. Whether the first law of thermodynamics holds on the horizons in different gravity theories have been studied generally (for example, see, [42,43,44]).

Now let us show that the first law of thermodynamics holds in the universe described by the present model. The amount of energy crossing the Hubble horizon during the time interval dt [41,45] is

(9) $$-dE=4\pi R_{h}^{2}T_{\mu \nu }k^{\mu }k^{\nu }dt=\frac{4\pi }{H^{2}}\left(\rho +p\right)dt,$$

where $R_{h}$ is the Hubble radius and $k^{\mu }$ is the future directed ingoing null vector field.

Using Equations (6) and (7), we obtain

(10) $$\rho +p=-\frac{\dot{H}}{4\pi L_{p}^{2}}\left(1+\frac{\dot{H}}{2H^{2}}\right),$$

so the amount of energy crossing the Hubble horizon during the infinitesimal time interval is expressed as

(11) $$-dE=-\frac{\dot{H}}{H^{2}L_{p}^{2}}\left(1+\frac{\dot{H}}{2H^{2}}\right)dt.$$

On the other hand, we can obtain

(12) $$TdS=-\frac{\dot{H}}{H^{2}L_{p}^{2}}\left(1+\frac{\dot{H}}{2H^{2}}\right)dt,$$

where we use the definition of temperature Equation (5) and the area-entropy relation S= $\frac{A}{4L_{p}^{2}}=\frac{\pi }{H^{2}L_{p}^{2}}$. Comparing Equations (11) with (12), we obtain the following equality

(13) −dE=TdS,

which implies that the first law of thermodynamics holds in the present model.

It is important to note that the strong energy condition ρ+3p≥0 is broken from Equation (4). However, we can see that the null energy condition ρ+p≥0 can be satisfied when $\dot{H}$ is nonpositive from Equation (10) because the term $1+\frac{\dot{H}}{2H^{2}}$ which is related with the temperature of the Hubble horizon is positive. In fact, it satisfies the GSLT for the accelerated universe which satisfies the null energy condition.

It is also worth mentioning that the amount of heat flux crossing the Hubble horizon during the infinitesimal time interval, δQ, is the change of the energy inside the Hubble horizon −dE. The minus appears due to the fact that the energy inside the Hubble horizon decreases when the heat flux flows out of the Hubble horizon, so the law (13) is actually Clausius relation δQ=TdS. Therefore, the evolution of the universe in the present model can be deemed as a series of quasistatic processes because Clausius relation works only when the thermodynamic process is reversible. Thus, the temperature of the matter inside the Hubble horizon can be taken as the temperature of the Hubble horizon. This is an important relation that we will use when we discuss the GSTL in the next section.

## 4. Validity of the GSLT and Thermodynamic Equilibrium

We have shown that the first law of thermodynamics holds on the horizon in the previous section; it is natural to ask if the GSLT holds in such a model. In the cosmological context, the GSLT denotes that the sum of the entropy of the cosmological horizon, $S_{h}$, plus the entropy of the matter inside the horizon, $S_{m}$, is a nondecreasing function. That is to say, the GSLT can be formulated as [31,32,46]

(14) $$\dot{S}={\dot{S}}_{m}+{\dot{S}}_{h}\geq 0.$$

Further, if the universe can reach an equilibrium state eventually, then the total entropy must satisfy the inequality

(15) $$\ddot{S}={\ddot{S}}_{m}+{\ddot{S}}_{h}\leq 0$$

at least at the last stage of evolution. The physical meaning of this inequality can be explained by the fact that the entropy of the universe increases less and less and reaches a maximum if the universe reaches an equilibrium state eventually.

Here we would like to point out that inequality (15) is slightly different from the one in the references [47,48] where the authors used the expression in which the second derivative of the total entropy is less than 0, i.e., $\ddot{S}={\ddot{S}}_{m}+{\ddot{S}}_{h}<0$. However, we allow the equal sign of the inequality (15) to be valid because it is possible for the total entropy to take the maximum value even if the first and second derivatives of the total entropy are both zero. In fact, the first and second derivatives of the total entropy are both zero when the universe evolves into the de Sitter universe.

According to the Gibbs relation and the conclusion of the previous section that the evolution of the universe can be deemed as a series of quasistatic processes, we know that the matter of the universe satisfies [34,35,49]

(16) $$TdS_{m}=d\left(\rho V\right)+pdV=\left(\rho +p\right)dV+Vd\rho ,$$

where $V=\frac{4\pi }{3H^{3}}$ is the Hubble volume and *T* equals to the temperature of the Hubble horizon. Substituting Equations (5), (7) and (10) into Equation (16), we obtain the change rate of the entropy of the matter

(17) $${\dot{S}}_{m}=\frac{2\pi }{H^{2}L_{p}^{2}\left(2H^{2}+\dot{H}\right)}\left[\frac{\ddot{H}}{2}+2H\dot{H}+\frac{{\dot{H}}^{3}}{H^{3}}+\frac{2{\dot{H}}^{2}}{H}\right].$$

For the Hubble horizon, the change rate of the entropy is

(18) $${\dot{S}}_{h}=-\frac{2\pi \dot{H}}{H^{3}L_{p}^{2}}.$$

Therefore, we can get the first derivative of the total entropy

(19) $$\dot{S}={\dot{S}}_{m}+{\dot{S}}_{h}=\frac{2\pi }{H^{3}L_{p}^{2}\left(2H^{2}+\dot{H}\right)}\left[\frac{\ddot{H}H}{2}+\frac{{\dot{H}}^{3}}{H^{2}}+{\dot{H}}^{2}\right]$$

and the second derivative of the total entropy

(20) $$\ddot{S}={\ddot{S}}_{m}+{\ddot{S}}_{h}=\frac{\pi }{L_{p}^{2}}\left[\frac{-34H^{2}{\dot{H}}^{4}-10{\dot{H}}^{5}+12H^{3}{\dot{H}}^{2}\ddot{H}+4H{\dot{H}}^{3}\ddot{H}+2H^{6}\dddot{H}-H^{4}\left(20{\dot{H}}^{3}+{\ddot{H}}^{2}-\dot{H}\dddot{H}\right)}{H^{6}\left(2H^{2}+\dot{H}\right)}\right].$$

Before we discuss the GSLT, let us obtain the constraints which are imposed on the energy density and pressure of the matter by the present model. Equation (10) can be transformed into

(21) $${\dot{H}}^{2}+2H^{2}\dot{H}+8\pi L_{p}^{2}H^{2}\left(\rho +p\right)=0.$$

Solving this equation, we obtain the solutions

(22) $$\dot{H}=-H^{2}\pm H\sqrt{H^{2}-8\pi L_{p}^{2}\left(\rho +p\right)},$$

which imply that the sum of the energy density and the pressure must satisfy the relation

(23) $$\rho +p\leq \frac{H^{2}}{8\pi L_{p}^{2}}.$$

This constraint gives the upper bound of the sum of the energy density and the pressure.

Now we investigate the GSLT in the present time and the last time of the evolution, respectively.

(i) The GSLT in the present time of the evolution. At the present time, we assume that the scale factor behaves as

(24) $$a\left(t\right)\propto t^{\alpha }$$

where α is a constant greater than unity because the universe is in accelerating expansion. This form can be obtained when the relation $8\pi L_{p}^{2}\left(\rho +p\right)\propto H^{2}$ is satisfied. In Ref. [36], it has been proven that the form of the scale factor is $a\left(t\right)=t^{\frac{2}{3(1+\omega )}}$ if the equation of state of the matter is assumed as p=ωρ where ω is a constant that is not equal to −1. In fact, a large number of papers on the accelerated expansion of the universe have assumed that the scale factor is the form (24). For example, the authors have pointed out that the rate of growth $a\left(t\right)\propto t^{2}$ is consistent with supernova observations in Ref. [50]. After some calculations, we obtain

(25) $$H=\frac{\alpha }{t},\;\dot{H}=-\frac{\alpha }{t^{2}},\;\ddot{H}=\frac{2\alpha }{t^{3}}.$$

Inserting Equation (25) into Equation (19), we obtain the change rate of the total entropy

(26) $$\dot{S}=\frac{2\pi t}{\alpha^{3}L_{p}^{2}}$$

which is greater than zero obviously, so the GSLT is satisfied in the present time of the evolution. The term related with the temperature of the Hubble horizon, $1+\frac{\dot{H}}{2H^{2}}$, could be derived from Equation (25) as $1-\frac{1}{2\alpha }$, which shows that the temperature of the Hubble horizon is positive for the current accelerated expansion of the universe (α>1). Further, we find that the null energy condition holds in the present time of the evolution because Equation (10) is positive.

(ii) The GSLT in the last time of the evolution. The Friedmann acceleration Equation (6) is derived from the fact that our universe is asymptotically de Sitter, so the scale factor $a\left(t\right)\to Ae^{H_{0}t}$ when time t→∞ where *A* and $H_{0}$ are both positive constants. Thus, the scale factor can be taken as

(27) $$a\left(t\right)\propto \sinh\left(H_{0}t\right).$$

Under this assumption, we obtain the following physical quantities

(28) $$H=\frac{\dot{a}}{a}=H_{0}\coth\left(H_{0}t\right)$$

and

(29) $$\dot{H}=-H_{0}^{2}{\mathrm{csch}}^{2}\left(H_{0}t\right),\;\ddot{H}=2H^{3}\coth\left(H_{0}t\right){\mathrm{csch}}^{2}\left(H_{0}t\right).$$

Inserting Equations (28) and (29) into Equation (19), we obtain

(30) $$\dot{S}=\frac{\pi \left(8\cosh\left(2H_{0}t\right)+\cosh\left(4H_{0}t\right)-1\right){\mathrm{sech}}^{4}\left(H_{0}t\right)\mathrm{sech}\left(2H_{0}t\right)\tanh\left(H_{0}t\right)}{4H_{0}L_{p}^{2}}\geq 0,$$

which implies that the total entropy is nondecreasing and the GSLT is satisfied. The derivative of the above expression, i.e., the second derivative of the total entropy is

(31) $$\ddot{S}=-\frac{\pi \left(54\cosh\left(2H_{0}t\right)-52\cosh\left(4H_{0}t\right)+10\cosh\left(6H_{0}t\right)+\cosh\left(8H_{0}t\right)-45\right){\mathrm{sech}}^{6}\left(H_{0}t\right){\mathrm{sech}}^{2}\left(2H_{0}t\right)}{16L_{p}^{2}}.$$

**Figure 1:**
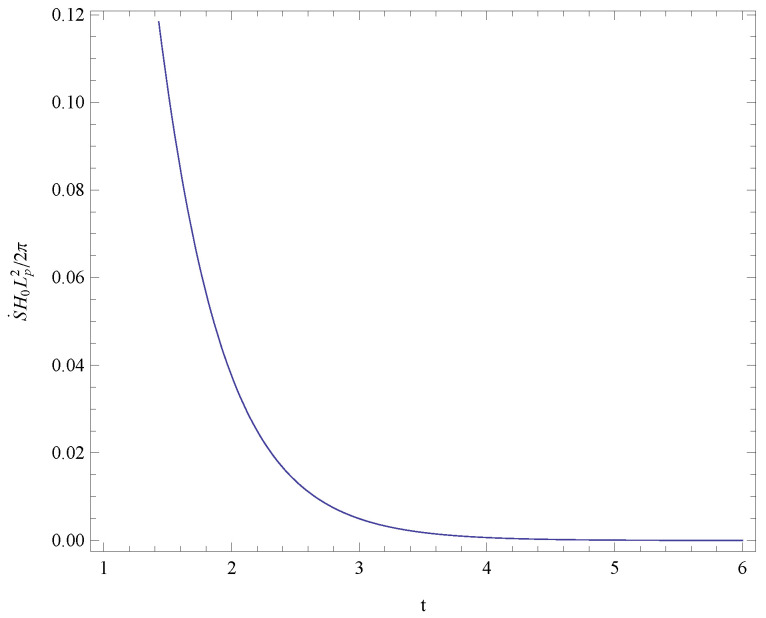
The variation of the first derivative of the total entropy in the time range of $1/H_{0}$ to $6/H_{0}$. This figure shows that the total entropy is nondecreasing and the GSLT (generalized second law of thermodynamics) is satisfied.

**Figure 2:**
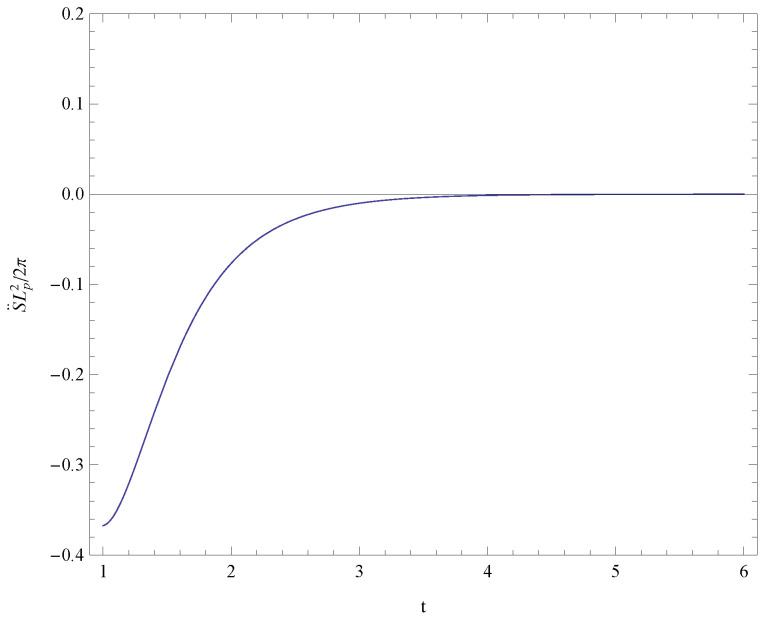
The variation of the second derivative of the total entropy in the time range of $1/H_{0}$ to $6/H_{0}$. This figure shows that the universe will tend to thermodynamic equilibrium for the sufficiently large time *t*.

Analyzing expression (31), we obtain the conclusion $\ddot{S}\leq 0$ for the sufficiently large time *t* which implies that the universe will tend to thermodynamic equilibrium. In order to see the conclusion $\dot{S}\geq 0$ and $\ddot{S}\leq 0$ clearly, we draw Figure 1 and Figure 2 to show the variation of the first and second derivatives of the total entropy in the time range of $1/H_{0}$ to $6/H_{0}$, respectively. The term related with the temperature of the Hubble horizon, $1+\frac{\dot{H}}{2H^{2}}$, could be derived from Equations (28) and (29) as $\frac{1}{2}\left[1+\tanh^{2}\left(H_{0}t\right)\right]$. This term is positive so the temperature of the Hubble horizon is positive. Further, we obtain the conclusion from Equation (10) that the null energy condition holds in the last time of the evolution.

At the end of this section, we investigate the special solution ρ+p=0 which depicts the de Sitter universe. When the equality ρ+p=0 is satisfied, we can obtain the solutions $\dot{H}=0$ or $\dot{H}=-2H^{2}$. For the solution $\dot{H}=-2H^{2}$, we see $a\left(t\right)\propto t^{1/2}$ which implies that the universe is not in accelerating expansion. This is inconsistent with the current assumption. Hence the unique solution is $\dot{H}=0$ which implies that *H* is a constant for the de Sitter universe. Substituting the solution $\dot{H}=0$ into Equation (19), we obtain $\dot{S}=0$ which implies that the GSLT is satisfied for the de Sitter universe. According to the above analysis, we conclude that the entire accelerated evolutionary process of the universe satisfies the GSLT and the total entropy of the universe tends to the maximal value which equals to the total entropy of the de Sitter universe in the present model.

## 5. Conclusions

In this paper, we study the first law of thermodynamics and the GSLT in the universe described by the emergence of space and the energy balance relation. First, we obtain the evolution equations of the universe based on the emergence of the space and the energy balance relation. In the process of derivation of the temperature of the Hubble horizon, we assume that the term $1+\dot{H}/H^{2}$ must be greater than zero. This assumption is reasonable because this term equals exactly to unity for the de Sitter universe and our universe is asymptotically de Sitter. Indeed, we show the validity of the assumption for the accelerated universe whose evolution law is $a\left(t\right)\propto t^{\alpha }$ or $a\left(t\right)\propto \sinh\left(H_{0}t\right)$ in Section 4. Next, we show that the first law of thermodynamics −dE=TdS is satisfied for the present model. In fact, the validity of the first law of thermodynamics implies that the Clausius relation δQ=TdS is satisfied in the present cosmological context. Therefore, the temperature of the matter inside the universe can been taken as the temperature of the Hubble horizon because the Clausius relation applies only to variations between the nearby states of local thermodynamic equilibrium.

Then, we analyze the GSLT and get the change rate of the total entropy according to the Gibbs relation and the area-entropy relation. Furthermore, we obtain the constraints which are imposed on the energy density and pressure of the matter by the present model. These constraints are $\rho +p\leq \frac{H^{2}}{8\pi L_{p}^{2}}$ and ρ+3p<0 respectively. To arrive at more specific results, we consider two typical examples in which the scale factor is taken as $a\left(t\right)\propto t^{\alpha }$ and $a\left(t\right)\propto \sinh\left(H_{0}t\right)$. The choice of the scale factor is based on the astronomical observation and the consistency with the current model. Whether the scale factor is taken as $a\left(t\right)\propto t^{\alpha }$ or $a\left(t\right)\propto \sinh\left(H_{0}t\right)$, the GSLT and these constraints are satisfied. At the same time, the null energy condition ρ+p≥0 is also satisfied. In addition, we find that the universe will reach a thermodynamic equilibrium state and the total entropy reaches a maximal value when time *t* tends to infinity. Hence we may conclude that there exists a good thermodynamic description for such a universe.

Finally, we must point out that these evolution equations have been obtained and the dynamical properties of such a universe have been studied in Ref. [36]. However, here we analyze the thermodynamic properties for this universe and find that the first law of thermodynamics and the GSLT are satisfied for two typical examples. The conclusions presented here further support the thermodynamic interpretation of gravity and reveal the connection between gravity and thermodynamics.

## References

[1] Riess A.G., Filippenko A.V., Challis P., Clocchiatti A., Diercks A., Garnavich P.M., Gilliland R.L., Hogan C.J., Jha S., Kirshner R.P. (1998). Observational Evidence from Supernovae for an Accelerating Universe and a Cosmological Constant. Astron. J..

[2] Perlmutter S., Aldering G., Goldhaber G., Knop R.A., Nugent P., Castro P.G., Deustua S., Fabbro S., Goobar A., Groom D.E. (1999). Measurements of Ω and Λ from 42 High-Redshift Supernovae. Astrophys. J..

[3] Padmanabhan T. (2003). Cosmological constant—The weight of the vacuum. Phys. Rept..

[4] Peebles P.J.E., Ratra B. (2003). The Cosmological constant and dark energy. Rev. Mod. Phys..

[5] Ratra B., Peebles P.J.E. (1988). Cosmological consequences of a rolling homogeneous scalar field. Phys. Rev. D.

[6] Ferreira P.G., Joyce M. (1997). Structure formation with a selftuning scalar field. Phys. Rev. Lett..

[7] Copeland E.J., Liddle A.R., Wands D. (1998). Exponential potentials and cosmological scaling solutions. Phys. Rev. D.

[8] Caldwell R.R., Dave R., Steinhardt P.J. (1998). Cosmological imprint of an energy component with general equation of state. Phys. Rev. Lett..

[9] Zlatev I., Wang L.M., Steinhardt P.J. (1999). Quintessence, cosmic coincidence, and the cosmological constant. Phys. Rev. Lett..

[10] Chiba T., Okabe T., Yamaguchi M. (2000). Kinetically driven quintessence. Phys. Rev. D.

[11] Armendariz-Picon C., Mukhanov V.F., Steinhardt P.J. (2000). A dynamical solution to the problem of a small cosmological constant and late time cosmic acceleration. Phys. Rev. Lett..

[12] Armendariz-Picon C., Mukhanov V.F., Steinhardt P.J. (2001). Essentials of *k*-essence. Phys. Rev. D.

[13] Gibbons G.W. (2002). Cosmological evolution of the rolling tachyon. Phys. Lett. B.

[14] Padmanabhan T. (2002). Accelerated expansion of the universe driven by tachyonic matter. Phys. Rev. D.

[15] Fairbairn M., Tytgat M.H.G. (2002). Inflation from a tachyon fluid?. Phy. Lett. B.

[16] Bagla J.S., Jassal H.K., Padmanabhan T. (2003). Cosmology with tachyon field as dark energy. Phys. Rev. D.

[17] Copeland E.J., Garousi M.R., Sami M., Tsujikawa S. (2005). What is needed of a tachyon if it is to be the dark energy?. Phys. Rev. D.

[18] Bardeen J.M., Carter B., Hawking S.W. (1973). The Four Laws of Black Hole Mechanics. Comm. Math. Phys..

[19] Bekenstein J.D. (1973). Black Holes and Entropy. Phys. Rev. D.

[20] Hawking S.W. (1975). Particle creation by black holes. Comm. Math. Phys..

[21] Maldacena J.M. (1998). The large *N* limit of superconformal field theories and supergravity. Adv. Theor. Math. Phys..

[22] Jacobson T. (1995). Thermodynamics of Spacetime: The Einstein Equation of State. Phys. Rev. Lett..

[23] Frolov A.V., Kofman L. (2003). Inflation and de Sitter thermodynamics. J. Cosmol. Astropart. Phys..

[24] Danielsson U.H. (2005). Transplanckian energy production and slow roll inflation. Phys. Rev. D.

[25] Padmanabhan T. (2002). Classical and quantum thermodynamics of horizons in spherically symmetric spacetimes. Class. Quan. Grav..

[26] Padmanabhan T. (2005). Gravity and the thermodynamics of horizons. Phys. Rep..

[27] Padmanabhan T. (2007). Gravity as an emergent phenomenon: A conceptual description. AIP Conf. Proc..

[28] Padmanabhan T. (2008). Gravity: the inside story. Gen. Rel. Grav..

[29] Padmanabhan T. (2012). Emergence and Expansion of Cosmic Space as due to the Quest for Holographic Equipartition. arXiv.

[30] Padmanabhan T. (2012). Emergent perspective of Gravity and Dark Energy. Res. Astron. Astrophys..

[31] Davies P.C.W. (1987). Cosmological horizons and the generalised second law of thermodynamics. Class. Quan. Grav..

[32] Davies P.C.W. (1988). Cosmological horizons and entropy. Class. Quan. Grav..

[33] Sadjadi H.M. (2006). Generalized second law in a phantom-dominated universe. Phys. Rev. D.

[34] Sheykhi A., Wang B. (2009). Generalized second law of thermodynamics in Gauss–Bonnet braneworld. Phys. Lett. B.

[35] Sheykhi A. (2010). Thermodynamics of apparent horizon and modified Friedmann equations. Eur. Phys. J. C.

[36] Tu F.-Q., Chen Y.-X., Sun B., Yang Y.-C. (2018). Accelerated expansion of the universe based on emergence of space and thermodynamics of the horizon. Phys. Lett. B.

[37] Gibbons G.W., Hawking S.W. (1977). Cosmological event horizons, thermodynamics, and particle creation. Phys. Rev. D.

[38] Cai R.-G., Kim S.P. (2005). First Law of Thermodynamics and Friedmann Equations of Friedmann-Robertson- Walker Universe. J. High Energy Phys..

[39] Calcagni G. (2005). de Sitter thermodynamics and the braneworld. J. High Energy Phys..

[40] Padmanabhan T. (2017). Do We really Understand the Cosmos?. Comptes Rendus Phys..

[41] Bousso R. (2005). Cosmology and the S matrix. Phys. Rev. D.

[42] Akbar M., Cai R.-G. (2006). Friedmann Equations of FRW Universe in Scalar–tensor Gravity, *f*(*R*) Gravity and First Law of Thermodynamics. Phys. Lett. B.

[43] Akbar M., Cai R.-G. (2007). Thermodynamic behavior of field equations for *f*(*R*) gravity. Phys. Lett. B.

[44] Wu S.-F., Wang B., Yang G.-H. (2008). Thermodynamics on the apparent horizon in generalized gravity theories. Nucl. Phys. B.

[45] Chakraborty S. (2012). Is thermodynamics of the universe bounded by event horizon a Bekenstein system?. Phys. Lett. B.

[46] Easther R., Lowe D. (1999). Holography, Cosmology, and the Second Law of Thermodynamics. Phys. Rev. Lett..

[47] Pavon D., Zimdahl W. (2012). A thermodynamic characterization of future singularities?. Phys. Lett. B.

[48] Saha S., Chakraborty S. (2012). A redefinition of Hawking temperature on the event horizon: Thermodynamical equilibrium. Phys. Lett. B.

[49] Izquierdo G., Pavon D. (2006). Dark energy and the generalized second law. Phys. Lett. B.

[50] Padmanabhan T., Choudhury T.R. (2002). Can the clustered dark matter and the smooth dark energy arise from the same scalar field?. Phys. Rev. D.

